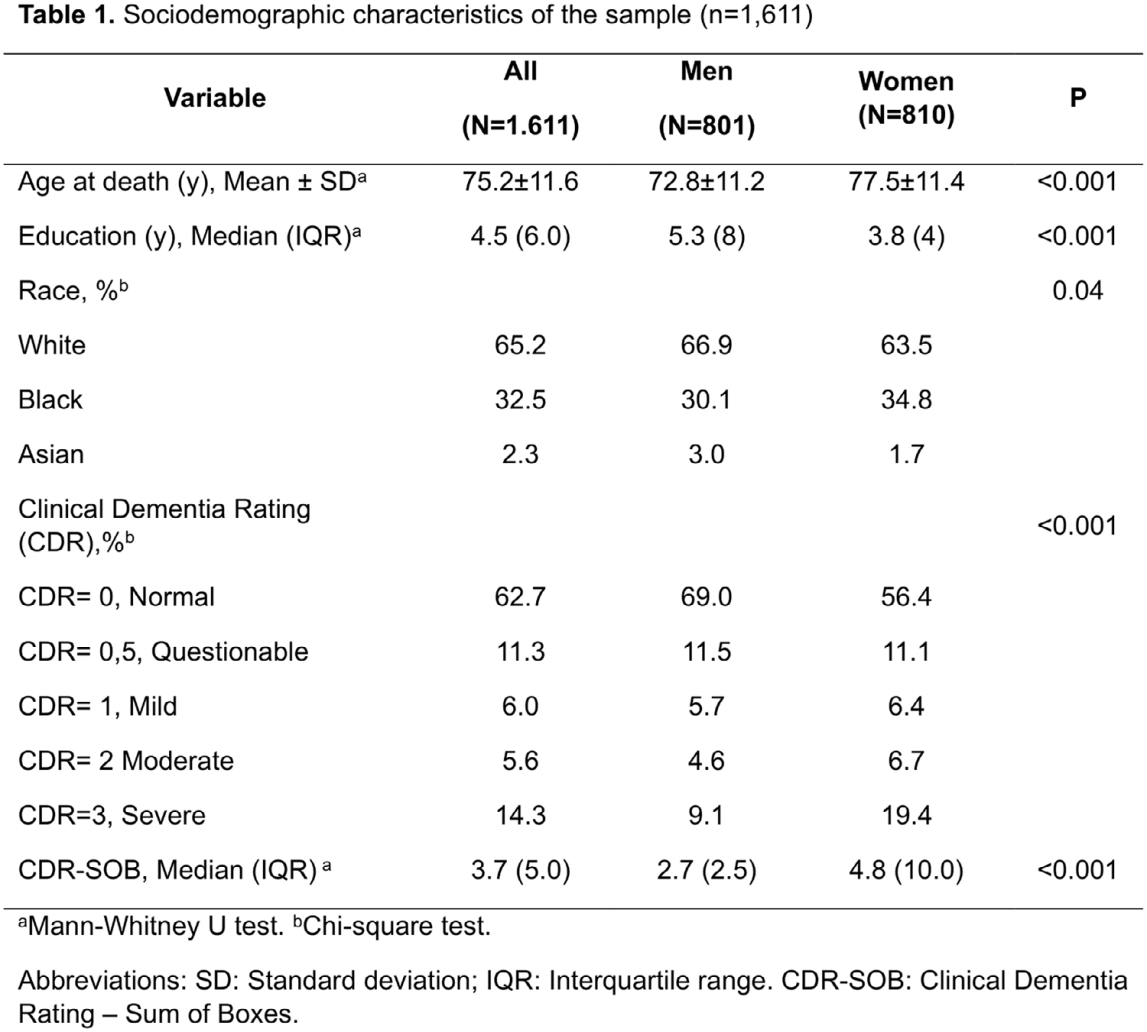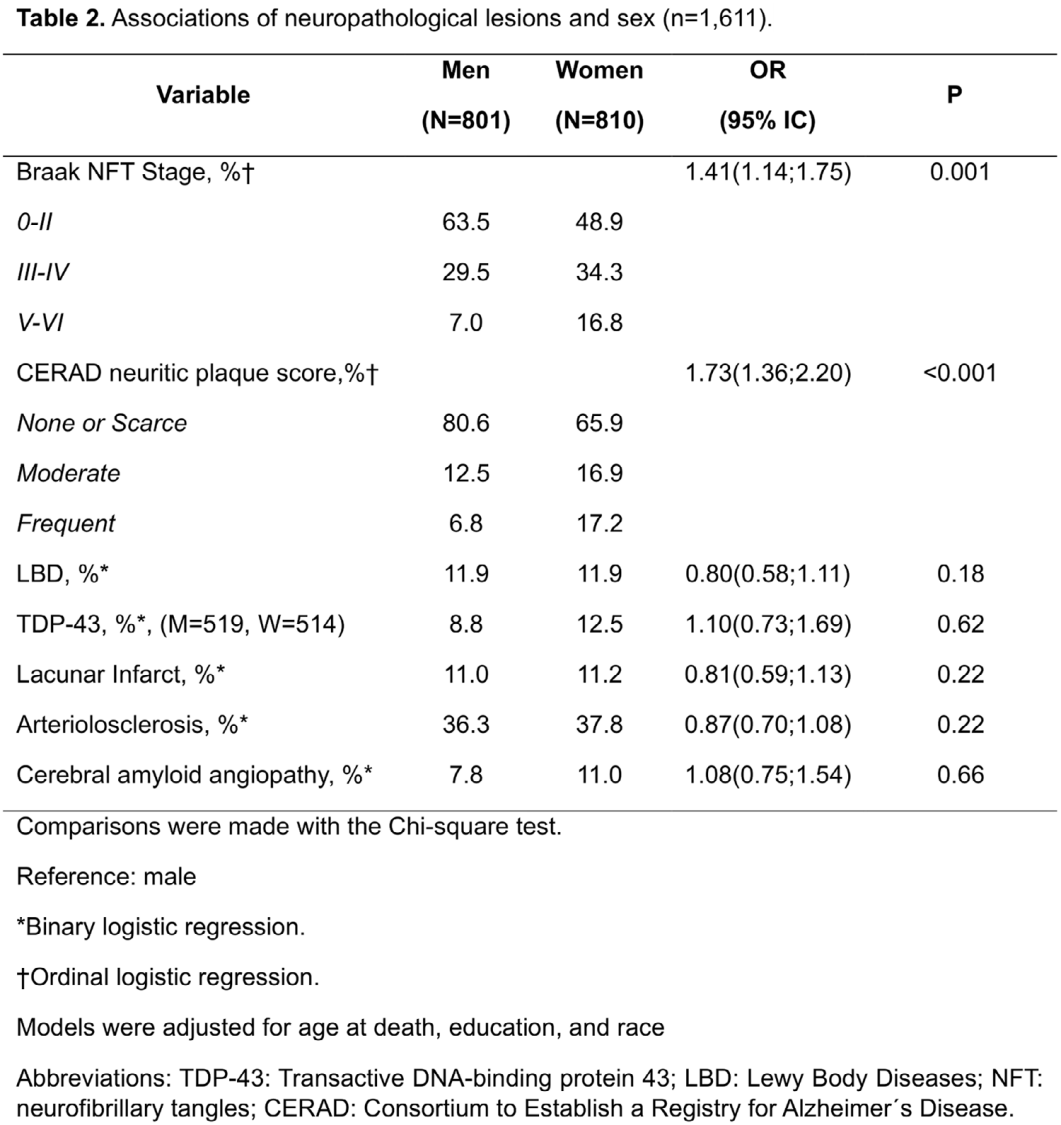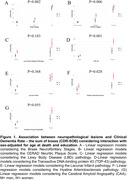# Clinical and neuropathological differences in dementia and the correlation between the sexes

**DOI:** 10.1002/alz.093088

**Published:** 2025-01-03

**Authors:** Karen L R Socher, Alberto Fernando Oliveira Justo, Roberta Diehl Rodriguez, Renata Elaine Paraizo Leite, Wilson Jacob‐Filho, Carlos Augusto Pasquallucci, Ricardo Nitrini, Lea T. Grinberg, Claudia Kimie Suemoto, Sonia Maria Dozzi Brucki

**Affiliations:** ^1^ University of São Paulo Medical School, São Paulo Brazil; ^2^ Hospital das Clinicas ‐ FMUSP, São Paulo Brazil; ^3^ Biobank for Aging Studies of the University of São Paulo, São Paulo Brazil; ^4^ University of São Paulo Medical School, São Paulo, São Paulo Brazil; ^5^ Physiopathology in Aging Laboratory (LIM‐22), University of Sao Paulo Medical School, São Paulo, São Paulo Brazil; ^6^ Cognitive and Behavioral Neurology Unit ‐ University of São Paulo, São Paulo Brazil; ^7^ LIM44, Departamento de Radiologia e Oncologia, Faculdade de Medicina da Universidade de São Paulo, Sao Paulo Brazil; ^8^ Biobank for aging studies of the University of São Paulo, São Paulo Brazil; ^9^ Faculdade de Medicina da Universidade de São Paulo, São Paulo Brazil; ^10^ University of São Paulo, São Paulo Brazil; ^11^ Universidade de Sao Pablo, Sao Pablo Brazil; ^12^ Brazilian Brain Bank of the Aging Brain Study Group; University of São Paulo, São Paulo Brazil; ^13^ Brain Bank of the Brazilian Brain Aging Study Group, São Paulo Brazil; ^14^ Memory & Aging Center, Department of Neurology, University of California in San Francisco, San Francisco, CA USA; ^15^ Division of Geriatrics, Department of Internal Medicine, University of Sao Paulo Medical School, São Paulo, São Paulo Brazil; ^16^ Hospital das Clínicas, Faculdade de Medicina da Universidade de São Paulo, São Paulo, São Paulo Brazil; ^17^ Medical School of University of São Paulo, São Paulo Brazil; ^18^ Cognitive and Behavioral Neurology Unit ‐ University of São Paulo, São Paulo, Brazil, Sao Paulo Brazil

## Abstract

**Background:**

The study of dementia and its differences between the sexes is widely investigated, mainly in Alzheimer’s disease. However, most studies on dementia are not carried out in a multiethnic population. Here we analyze demographic data, clinical symptoms, and neuropathological characteristics of a large mixed Brazilian sample.

**Method:**

The Biobank for Aging Studies has a unique collection of brain samples that we selected over 50 years of age. All were examined for the presence of dementia pathology according to internationally accepted criteria for pathological staging and diagnosis. All cases have a complete semi‐structured interview carried out to obtain sociodemographic data, lifestyle, past medical history, and the presence of cognitive impairment (CI) was defined according to the Clinical Dementia Rating Scale (CDR≥0.5).

**Result:**

Of the 1,611 cases included, 801 were men and 811 were women. Regarding ethnicity, 65.2% were identified by family members as white for both groups. Regarding differences between sexes, women were older, less literate and had greater cognitive impairment in the CDR and Sum of Boxes (p>0.001). The neuropathological findings showed a sample of women with a lower percentage (48.9%) of initial Braak stages and a higher percentage (51.3%) of Braak stage ≥III (p>0.001). Higher prevalence of moderate and frequent CERAD score (p>0.001), as well as TDP‐43 proteinopathy. Concerning other pathologies such as Lewy Body Diseases, Lacunar Infarction, Arteriosclerosis, and Cerebral Amyloid Angiopathy, no significance was found. When analyzing sex and clinical symptoms using a multivariate linear regression model, there was significance for both the final CDR score and the sum of the boxes. The association of neuropathological and clinical findings through logistic regression with adjustment for age and education, there is a tendency for women to present more symptoms, regardless of the subtype of neuropathological lesion. For Alzheimer’s disease pathology, there was statistical significance with the clinical pathology and continuous staging with prevalence in women, as well as in the presence of TDP‐43 pathology and hyaline arteriosclerosis in vascular pathology.

**Conclusion:**

Sex in the neuropathology of dementia plays an important role in cognitive symptoms, highlighting the need to consider it in future prevention and treatment strategies.